# Optimizing Feeding and Pupation Bioassays to Assess the Effects of Insecticidal and Repellent Treatments on *Aethina tumida* Larval Development and Pupation Success

**DOI:** 10.1002/arch.70023

**Published:** 2025-01-21

**Authors:** Morgan A. Roth, Aaron D. Gross

**Affiliations:** ^1^ Molecular Physiology and Toxicology Laboratory, Department of Entomology Virginia Polytechnic Institute and State University Blacksburg Virginia USA

**Keywords:** amphotericin B, bioassay, insecticide, organophosphates, pupation, pyrethroids

## Abstract

European honey bee (*Apis mellifera*) colonies are an ideal host to the invasive beetle *Aethina tumida*, providing a nutrient rich environment that is protected from the elements and facilitates beetle reproduction. Although various management techniques and chemical treatments for *A. tumida* have been developed, understanding the efficacy of these treatments and techniques is limited. Throughout this study, several methods for impairing *A. tumida* development and delivering insecticidal, repellent, or antifungal treatments were examined. A series of *A. tumida* larval feeding bioassays developed and optimized feeding gel pellet for delivery of insecticidal treatments, revealing that *A. tumida* larvae are sensitive to the two common in‐hive varroa mite (*Varroa destructor*) treatments: coumaphos (EC_50_ = 25.6 ppm) and *tau*‐fluvalinate (EC_50_ = 21.2 ppm). Feeding bioassays also demonstrated that *A. tumida* were more sensitive to the pyrethroid compounds permethrin (EC_50_ = 3.37 ppm), deltamethrin (EC_50_ = 2.69 ppm), and bifenthrin (EC_50_ = 0.365 ppm), which have been previously used to control this beetle. Feeding bioassays also revealed that the antifungal drug Amphotericin B was palatable to *A. tumida* larvae via feeding, but was also injected into *A. tumida* larvae and adults. Two types of pupation bioassays were also developed to test the effects of several insecticidal and repellent treatments on pupation burrowing and pupation success. Overall, this work details specific toxicity information regarding common insecticidal treatments found in the apiary setting study and provides groundwork and methods for testing insecticidal compounds on *A. tumida* larvae in in the future.

## Summary

- Development of a feeding bioassay with an agarose pellet containing 25% sugar and 2% soy protein can be used to test insecticides and fungicides.
- Amphotericin B does not eliminate *Kodamea ohmeri*.
- Coumaphos that is commonly used to control varroa mites is not as effective against small hive beetles.
- Current *Varroa destructor* treatments are not as effective against small hive beetles.

## Graphical abstract

The small hive beetle larvae are a destructive pest that affects honey bee hives. It has limited effective control methods. This manuscript describes bioassays developed to evaluate toxic molecules orally given or in contact with the soil.

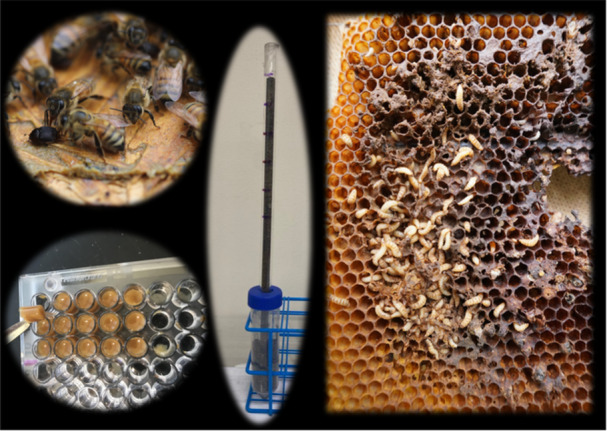

## 1 Introduction

*Aethina tumida* have been invading *Apis mellifera* hives in the United States since 1998 (Elzen et al. 1999), but consistently effective management options have remained somewhat limited throughout this time, though not for lack of effort. There are several key difficulties that make targeting *A. tumida* a complicated task, the first of which is that any treatment used within the hive environment has the potential to harm the *A. mellifera* colonies being treated. Thus, one of the most popular management methods to date is trapping, which mostly targets adults and only reduces adult numbers (Hood 2011). Although reducing adult beetle populations in the apiary environment is unequivocally beneficial, *A. tumida* larvae are responsible for most of the hive damage incurred by *A. tumida* infestations (Hayes et al. 2015). One *A. tumida* female can lay 1000–2000 eggs, so a few adult beetles can still generate numerous larvae (Somerville 2003).

Despite the potential risks of chemical treatments, there are several synthetic chemicals that are commonly used in apiary settings in the context of *Varroa destructor* control, including the octopamine receptor agonist amitraz (Blenau, Rademacher, and Baumann 2012), the organophosphate and acetylcholinesterase inhibitor coumaphos (Elzen, Westervelt, and Lucas 2004), and a sodium channel modulator and pyrethroid *tau*‐fluvalinate (Baxter et al. 1998). As early as 1999, coumaphos was evaluated and recommended for use in the management of *A. tumida* (Elzen et al. 1999), but it is now known that coumaphos can have numerous detrimental impacts on *A. mellifera* colonies, ranging from reproductive damage to both queens and drones (Chaimanee et al. 2016), lifespan reduction (Wu, Anelli, and Sheppard 2011), and olfactory impairment (Williamson, Baker, and Wright 2013) to buildup in wax over time (Premrov Bajuk et al. 2017). Other chemical treatments that have been used exclusively for *A. tumida* management are pyrethroids, the most popular of which is permethrin (GuardStar), which has been used in soil drenching (Hood 2004). However, pyrethroids can also be toxic to honey bees, causing a host of health issues, including reproductive system impacts, reduction in foraging, learning, and olfactory functions (Decourtye et al. 2004), which is likely the result of the suppressed neuronal activity that results from pyrethroid exposure (Zhou et al. 2011).

The *A. tumida* lifecycle takes place both inside the hive (eggs and larval development) and outside of the hive (pupation and emergent adults) (Neumann, Pettis, and Schäfer 2016). A further management complication is added by the fact that *A. tumida* adults are ultimately scavengers and can survive and reproduce on a variety of fruits (Buchholz et al. 2008), as well as in the hives of other bee species, including *Bombus impatiens* (Graham et al. 2011) and several species of melponine bees (Bobadoye et al. 2018). Since *A. tumida* populations can thrive outside of the managed apiary environment, it is virtually impossible to eliminate *A. tumida* from a geographic area. Additionally, there is not an established *A. tumida* economic threshold to inform beekeepers regarding appropriate treatment measures to incorporate based on infestation severity. Because *Varroa destructor* is such a disastrous hive pest, resources and research have been highly focused on management for this pest, which *A. tumida* has received relatively little attention. Therefore, there are many aspects of *A. tumida* biology and management options that are yet to be explored.

One of the most fascinating aspects of *A. tumida* infestations is the fungal symbiont *Kodamea ohmeri* (Amos et al. 2018), which is an ascomycetous yeast that is closely related to *Candida albicans* (Leemon 2012). Although limited work has been done examining the importance of *K. ohmeri* to *A. tumida* growth and development, this fungus has been isolated from each *A. tumida* life stage, starting in the mucilage of the eggs to external surfaces and the digestive tracts of adult beetles (Amos et al. 2018). It has been suggested that *K. ohmeri* is merely a facultative symbiont and is unnecessary for the successful completion of the *A. tumida* lifecycle, however, this study was not carried out for more than one generation and was performed under laboratory conditions (Amos et al. 2019). Even though *K. ohmeri* may simply be a facultative fungal symbiont, its prevalence throughout the *A. tumida* lifecycle and its role in facilitating the breakdown of hive products and subsequent production of volatiles that are attractive to *A. tumida* adults (Benda et al. 2008), suggests that further steps could be taken to test repression of this fungus to better determine the value of *K. ohmeri* to the *A. tumida* lifecycle.

In view of the lack of knowledge pertaining to *A. tumida* chemical treatments, along with the need for clearer insights regarding the function of *K. ohmeri* throughout the *A. tumida* lifecycle, the following study was developed. The goal of this work was to establish effective methods for *A. tumida* feeding and soil bioassays and to understand whether or not fungicide feeding could impact internal *K. ohmeri* presence and detrimentally affect *A. tumida* development and survival. The first step in this work was to develop a feeding bioassay that both sustained larval development and was an effective means of drug delivery. Building upon the successful feeding bioassays developed, a series of experiments testing the effects of the fungicide Amphotericin B were carried out. Amphotericin B is known to inhibit *K. ohmeri* and has been principally used in human medicine in cases of fungal infection (Kanno et al. 2017). Injection of Amphotericin B into third instar *A. tumida* larvae and subsequent *K. ohmeri* tests were also performed. Finally, two types of soil toxicity bioassays were developed, with one bioassay focused on pupation depth and the other focused on pupation success. These bioassays tested coumaphos and permethrin, along with several repellent compounds, to determine whether or not such treatments could deter larvae from burrowing and pupating.

## 2 Materials and Methods

### 2.1 Chemicals

Amitraz (> 98%), bifenthrin (99%), deltamethrin (> 99%), and permethrin (47.6% cis; 50.4% trans) were all purchased from Chem Service Inc. (West Chester, PA, USA). Coumaphos (99%), *tau*‐fluvalinate (98.7%), pyrrole (98%), pyrrolidine (≥ 99.0%), amphotericin B (solubilized, suitable for cell culture), and potassium chloride (≥ 99.0%) were all purchased from Sigma‐Aldrich (St. Louis, MO, USA). Sec‐butyl 2‐(2‐hydroxyethyl) piperidine‐1‐carboxylate (Picaridin; 98%) was purchased from AmBeed (Arlington Heights, IL, USA). Sodium chloride (≥ 99.0%), HEPES (≥ 99.0%), calcium chloride dihydrate (≥ 99.0%), Difco Sabouraud Dextrose Broth, and the acetone (HPLC grade) used for dilution of all compounds in this study was from Fisher Scientific (Hampton, NH, USA). AP23 (Artificial Pollen) patties were purchased from Dadant and Sons Inc. (Hamilton, IL, USA). All granulated sugar (99% sucrose) used was from the Smidge and Spoon brand (Novi, MI, USA). Soy protein (isolated)was purchased from MP Biomedicals LLC (Solon, OH, USA). Apex Bioresearch Products Agarose (General Purpose LE) and Apex chloramphenicol were purchased from Genesee Scientific (San Diego, CA, USA). 4e.

### 2.2 Insects

*A. tumida* adults were collected from the Prices Fork apiary in Blacksburg, VA, USA in August of 2018 and were used to start a laboratory colony. A specimen from the laboratory colony has been added to the Virginia Tech collection (Catalogue Number: VTEC000004965).

Rearing was performed as previously stated (Roth, Lahondère, and Gross 2023), with minor modifications. The colony was maintained in a dark environmental chamber (Thermo Scientific, Waltham, MA, USA) at 28°C and greater than 70% relative humidity. Larvae were feed on pollen patty until they reach the third‐instar when they were placed into a pupation conical (50 mL) that was composed of approximately 30 third‐instar larvae, moistened 1:1 sand/soil. The pupation conical was covered with parafilm containing small ventilation holes. Larvae often burrow down the sides of pupation conical tubes, where they could be visualized to monitor adult emergence. When visible adults had emerged, the conical tubes were left for several days to allow the cuticles of the newly emerged adults to sclerotize, then the adults were added to an adult rearing container of the appropriate generation.

### 2.3 Feeding Bioassay Development

To carry out feeding bioassays, the first step was the optimization of feeding pellets. Although ground pollen granules were first tested in the formulation of food pellets, these granules were very large and, even after being frozen and ground to a fine powder with a mortar and pestle, did not readily disperse in agarose mixtures. Thus, tests with soy protein powder in agarose were carried out, along with tests of various sugar concentrations in agarose, to determine optimum nutrient ratios to keep larvae alive. Sugar concentrations at 10%, 20%, 30%, 40%, and 50% were tested, along with positive controls of pollen patty and agarose pellets with no sugar or protein. Soy protein was tested using concentrations of 1.5%, 3%, 6%, 9%, and 12%, at which point the powder became too thick to dissolve into the agarose. To make these pellets, 50 mL of a 1% agarose solution was made in a 250 mL Erlenmeyer flask and appropriate volumes of either sugar or soy protein were added while the solution was still above 65°C. The agarose solutions were added to wells of a flat‐bottomed 96‐well microtiter plate, with each well holding approximately 300 μL of the agarose mixture. Once the agarose mixture in the wells solidified, they were dislodged and retained their cylindrical shape. Based upon preliminary studies, the optimum pellet formulation contained 25% sugar and 2% soy protein solution (Figure S1a). Insecticide and drug treatments were added to this sugar and protein concentration, while in the liquid form, and the treatments were vortexed before making feeding pellets, as described above.

Five late first instar/early second instar *A. tumida* larvae were added to 2 oz clear plastic souffle/portion cups, with a small square of mesh glued to an approximately 1‐cm^2^ hole in the lid for ventilation. Before the start of experiments, the average weights of each container of larvae were obtained, as was done at the end of all feeding bioassays. The experiments ended when larvae reached the late third instar stage, which was easily discernable, visually, and was generally reached 7–10 days after the experiment commenced. A minimum of three replicates were performed for each test compound.

### 2.4 Antifungal Feeding and Injection Bioassays

The first test carried out using amphotericin B treatments was a test of feeding effects upon *A. tumida* larval development. Because *A. tumida* instar can be determined based upon head capsule width, five first instar larvae per treatment were obtained and head capsule width was measured every other day for 7 days atop a chilled aluminum block. *A. tumida* first instar head capsules range from 250 to 350 μm, while head capsule widths range from 490 to 670 μm for second instar larvae, and 790–1170 μm for third instar larvae (Amos et al. 2018). Treatments for this bioassay consisted of a pollen patty control, an amphotericin B pellet (1 ppm), and a pellet containing the vehicle control (0.1% DMSO). Additionally, two sets of larvae were only fed on treated pellets for 24 h and then switched back to pollen patty for the remainder of the experiment to better understand the effects of limited fungicide feeding.

The second set of experiments fungicide feeding tests were performed by feeding second instar larvae pollen patty, agarose pellets, 1‐ppm Amphotericin B and 0.1% DMSO (negative control) pellets for 24 h, after which larvae were surface sterilized in a laminar flow hood following the methods of Amos et al. (2018). Briefly, larvae were submerged for 30 s at a time in 70% ethanol, sterile water, 5% bleach in aqueous polysorbate (Tween 80, 0.25%), and finally two rinses of sterile water, after which they were placed in a 1.7 mL Eppendorf tube containing 250 μL of sterile water. A plastic pestle and mechanical mortar were then used to homogenize the larva and the homogenate was diluted fivefold before spreading 50 μL on a Petri dish of Sabouraud's Dextrose Agar (SDA). *Kodamaea ohmeri* is known to successfully grow on SDA and can easily be morphologically identified (Leemon 2012) and 0.5 mg/mL of chloramphenicol is also added to the SDA to inhibit bacterial growth. After spreading homogenate, plates were incubated in the dark and images obtained after 48‐ and 72‐h intervals using an iPhone 11 camera beneath a single light source. Colonies were quantified using ImageJ software (www.imagej.nih.gov), which allowed conversion of color photos to 8‐bit grayscale images. Colonies were then quantified and outlined under circularity settings of 0.30–1.00, with edges excluded.

Fungicide feeding was also carried out with adult beetles. Before feeding, adults were surface sterilized and then fed for 2 days on each of the treatments described above. Adults then were all fed upon pollen patty for the next 10–12 days and egg laying was monitored over 5 days. The adult beetles were then surface sterilized again, homogenized, and plated, and colonies were quantified as described above. Eggs were also counted over this time period.

The next *K. ohmeri* repression experiments were performed by modifying the delivery system to melted pollen patty, as the goal was to try to feed larvae on an amphotericin B treatment for the entirety of their development. To accomplish this, pollen patty was melted into a 0.1% agarose solution and larger agarose disks were created using 3.5‐cm Petri dishes. Similar feeding disks were made using 0.1% DMSO and 2 ppm amphotericin B. Glass microscope slides containing *A. tumida* eggs were added to jars with each treatment and larvae were then fed to the third instar, after which internal *K. ohmeri* colony counts were quantified per the above methods. In a follow up experiment, amphotericin B levels were raised to a range of 10–1000 ppm, which was again fed to test larvae throughout the entirety of their development. Higher homogenate dilutions (10, 25, and 50‐fold) were also tested using these third instar larvae. Several untreated wild‐caught *A. tumida* adults and larvae were also compared lab‐reared adults and larvae, at homogenate dilutions of 25 and 50‐fold.

Finally, *A. tumida* injections were performed to test the delivery of 50 nL of 1 ppm amphotericin B, 0.1% DMSO, and CNS buffer control (157 mM NaCl, 3 mM KCl, 2 mM CaCl_2_, 4 mM HEPES) using a nanoliter injector (World Precision Instruments, Sarasota, FL, USA). After each injection, 50 nL of treatment was expelled onto a Kimwipe to ensure that the needle had not become blocked. Ten second instar larvae were tested per treatment and mortality was assessed after 2 days when all larvae had reached their third instar. Additionally, injected second instar larvae were homogenized and tested for internal *K*. *ohmeri* counts after treatment delivery and compared with third instar larvae that had survived for at least 2 days post injection.

### 2.5 Pupation Depth Bioassays

To test effects of insecticide treatments on pupation depth, 12 in. glass tubes were procured, inches were marked out on the tubes, and it was found that 1 in. of the tube held approximately 1.4 g of autoclaved 1:1 sand/soil mix. A 50 mL conical was used to form a base for each tube, with a hole made by a Dremel in the top of the conical lid to allow the tube to glass fit tightly. The inside of the conical was lightly sprayed with water to provide moister and prevent desiccation, the conical was then filled approximately 2/3 full. The glass tube was then inserted into the soil and another pump (spray bottle) of water was added before the conical was closed. The glass tube was next filled with the 1:1 sand/soil mixture up to the three‐inch mark (no additional water added). All treatments were made by weighing out 1.4 g of sand/soil (Figure S1b). One milliliter of each treatment (and 1 mL acetone control) was pipetted (500 μL at a time) on to the sand/soil, which was moved using the foil to help coat sand and soil particles more thoroughly. Once all treatments were administered and the sand/soil was dry, it was poured into the glass tube, and another inch of sand/soil was added over the treatment. A single‐third instar larva was then added to each tube (five tubes per replicate), and the tops of the tubes were covered in parafilm, with a single small hole added for ventilation. All tubes were then placed in a 50 mL conical holder and incubated at 28°C until pupae were observed on the sides of the tubes. Because the tubes were so narrow, visualization of pupae was not difficult. As the tubes were empty, the pupation depth of each larva was noted and measured.

### 2.6 Pupation Success Bioassays

Pupation success bioassays were developed to assess whether or not larvae would burrow past a treated range and whether or not pupation could successfully occur after passage through the treatment. Conical tubes (50 mL) were used for this experiment and centimeter increments were marked out on the sides of the tubes. It was found that a 1‐cm conical depth equated to approximately 5 g of sand/soil and 2 mL of each treatment was prepared and added to the sand/soil mixture on aluminum foil beneath a fume hood. The 2 mL treatment (and acetone control) saturated the five grams of soil, and agitation of the foil (side‐to‐side) helped move the sand/soil to help ensure that it was well coated in treatment. The 50 mL conicals were dampened using a spray bottle and sand/soil was added up to 3‐cm below the top of the conical. Once the treated sand/soil was dry, it was added to the conical, and 1‐cm of clean sand/soil was added on top of this. Twenty third‐instar larvae were added to each conical and parafilm was used to cover the conical tops, with several small holes added for ventilation. All conicals were kept incubated at 28°C until pupae could be observed along the sides of the conicals (approximately 2 weeks), after which they were emptied and larvae/pupae above, within, and below the treatment were counted and mortality was noted.

## 3 Results

### 3.1 Feeding Bioassays

The results of sugar feeding pellets showed that there was 100% survival in both replicates at the 20%, 40%, and 50% concentrations, however, average weight gain in all treatments was far lower than weight gain observed with pollen patty feeding, with an approximately 8‐mg difference between sugar treatments and pollen patty (Table 1). With the feeding pellet made solely of agarose, 40% mortality and weight loss were observed, indicating that this pellet was not a viable source of nutrition. Overall, there was no marked difference between weight gain, with overall average gain of 1.88 mg (10%), 1.4 mg (20%), 2.9 mg (30%), 1.5 mg (40%), and 2.2 mg (50%). Interestingly, the highest average weight gain and mortality (30%) occurred with the 30% sugar treatment. This test indicated that as long as a sugar source was present, the concentration did not greatly influence larval survivorship or growth.

**Table 1:** Results of sugar feeding bioassays (two reps combined).

Food	Avg. start weight (mg)	Avg. % mortality	Avg. end weight (mg)	Avg. weight change (mg)
Agarose	5.3	40	4.59	−0.71
Pollen patty	3.7	10	14.3	+10.6
10% sugar	4.5	10	6.38	+1.88
20% sugar	3.8	0	5.2	+1.4
30% sugar	4	30	6.9	+2.9
40% sugar	3.9	0	5.4	+1.5
50% sugar	3.9	0	6.1	+2.2

The results of the soy protein feeding bioassays told a different story. Once again, larvae thrived on the pollen patty positive control and languished with 80% mortality in the agarose negative control. Although average weight gain for each protein treatment was again much lower (1.2–1.9 mg) than average weight gain for pollen patty fed larvae (13.3 mg; Table 2), survivorship was also quite low with average mortalities ranging from 87% to 93% for all soy protein treatments. Again, it appeared that regardless of soy protein concentration, mortality and weight gain did not greatly differ, and a carbohydrate source was imperative for beetle survival and growth.

**Table 2:** Results of soy protein feeding bioassays (three reps combined).

Food	Avg. start weight (mg)	Avg. % mortality	Avg. end weight (mg)	Avg. weight change (mg)
Agarose	1.4	80	4.5	+3.1
Pollen patty	1.2	17	14.9	+13.7
1.5% protein	1.3	87	2.5	+1.2
3% protein	1.1	93	3	+1.9
6% protein	1.3	93	3	+1.7
9% protein	1.4	87	3	+1.6
12% protein	1.3	87	2.5	+1.2

Based upon the sugar and soy protein information, an agarose pellet containing 25% sugar and 2% soy protein was tested and used for all insecticide and feeding bioassays. At these sugar/protein ratios, control larvae thrived and reached the third instar, and larval development was comparative to that observed with pollen patty feeding. Of the six insecticides tested, the most potent oral half maximal‐lethal concentration (LC_50_) resulted from bifenthrin feeding (LC_50_ = 0.365 ppm) followed by deltamethrin (LC_50_ = 2.69 ppm), permethrin (LC_50_ = 3.47 ppm), and *tau*‐fluvalinate (LC_50_ = 21.2 ppm) (Table 3). The common in‐hive treatment coumaphos was only slightly less potent than *tau*‐fluvalinate, with an LC_50_ of 25.6 ppm (Table 3). An LC_50_ value could not be established for amitraz, with high larval survival at concentrations of 100 ppm (Table 3).

**Table 3:** Feeding bioassay toxicity data (three to five reps per treatment).

Compound	LC_50_ (ppm)	95% confidence interval	*n*	Rep #
Amitraz	> 100	—	60	3
Coumapho	25.6	20.2–30.7	150	5
*tau*‐fluvalinate	21.2	13.3–30.3	95	3
Bifenthrin	0.365	0.226–0.519	150	5
Deltamethrin	2.69	1.30–4.80	90	3
Permethrin	3.47	2.67–4.45	90	3

### 3.2 Antifungal Feeding and Injection Bioassays

**Figure 1:**
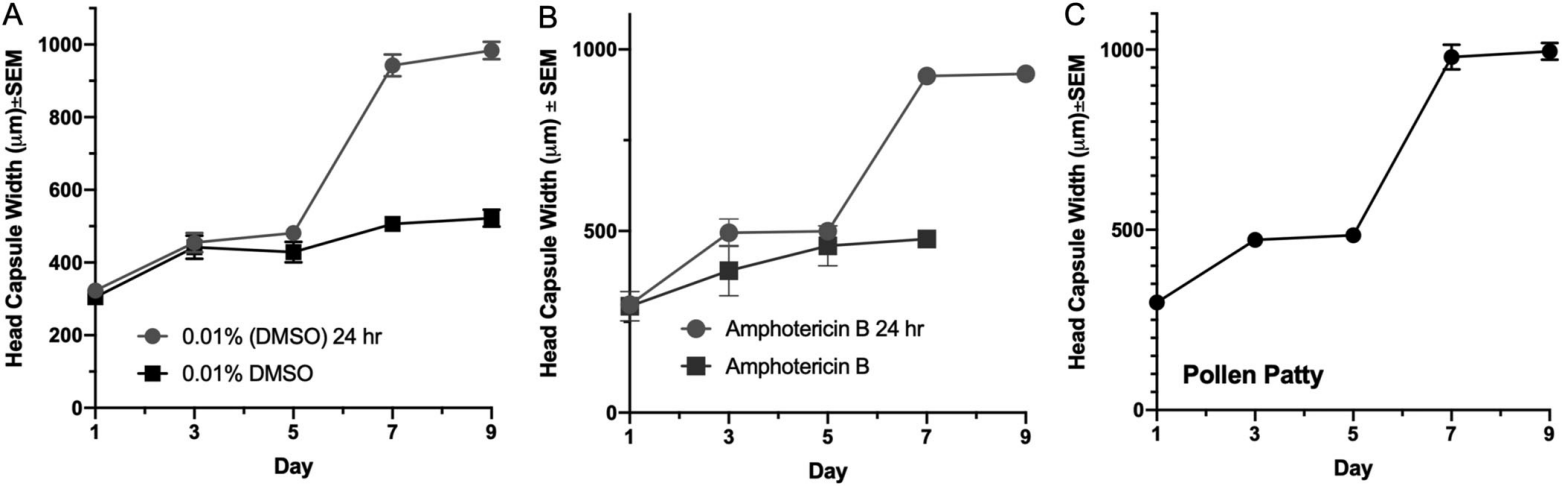
*Aethina tumida* growth based on head capsule width (first instar = 230–350 μm; second instar = 490–670 μm; third instar = 790–1170 μm) measured every other day over a 9‐day test period. Treatments consist of 0.1% DMSO until Day 9 and 0.1% DMSO feeding for 24 h followed by pollen patty feeding until Day 9 (A), 9‐day amphotericin B feeding and 24 h amphotericin B feeding followed by pollen patty feeding until day none (B), and pollen patty feeding from Days 1 thru 9 (C).

The effects of the amphotericin B on larval growth measured by head capsule width revealed that all larvae reached second‐instar head capsule widths by Day 3. Growth trends appeared similar for all insects until Day 7, at which time the pollen patty fed larvae and the larvae that had only been fed upon test compounds for 24 h reached their third‐instar, while larvae fed on the treatments through the entirety of the experiment remained in their second‐instar until Day 9 (0.1% DMSO) or died before Day 9 (1 ppm amphotericin B; Figure 1).

**Figure 2:**
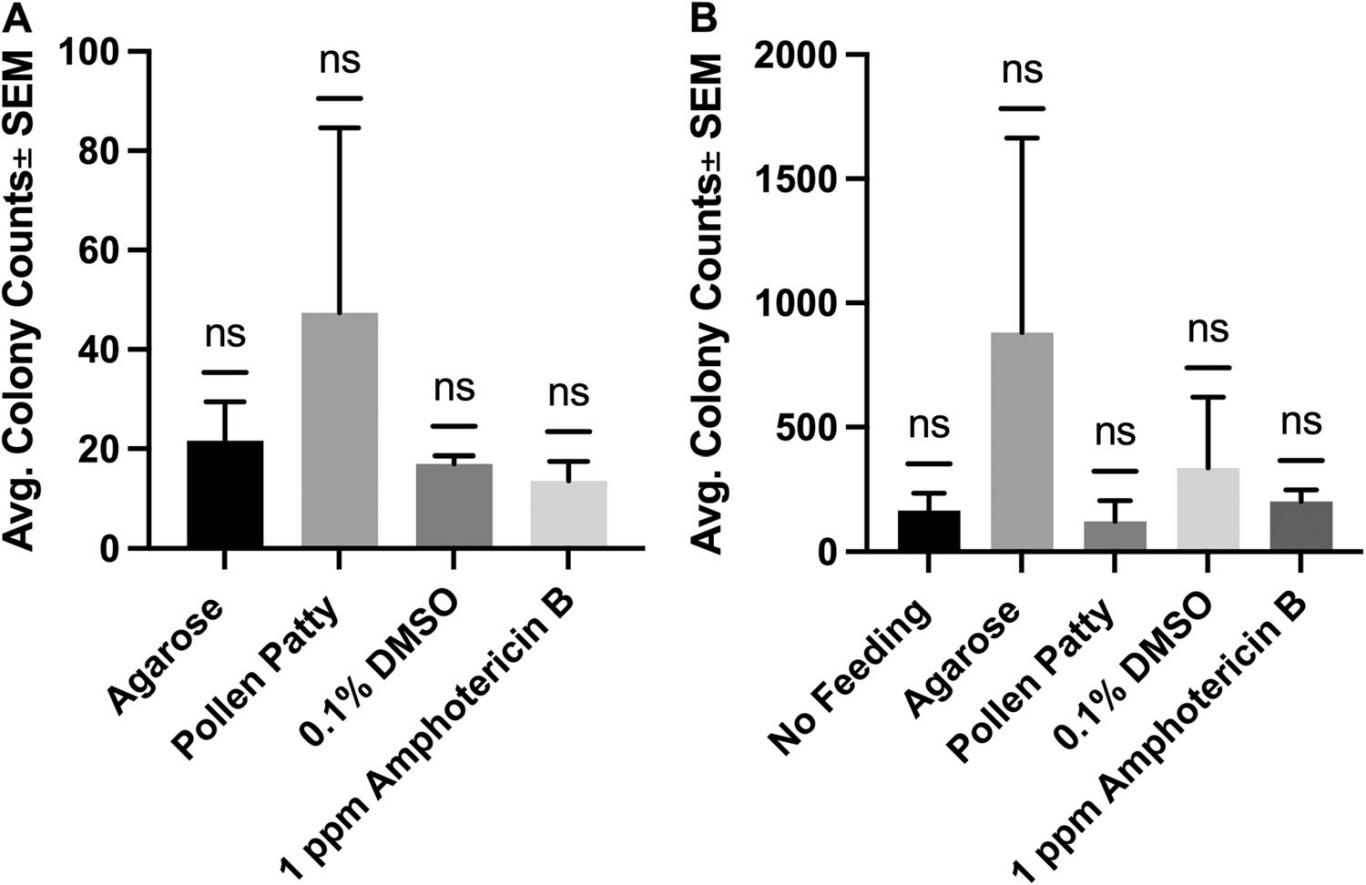
*Kodamaea ohmeri* colony counts 48 h post‐incubation resulting from *Aethina tumida* amphotericin B feeding tests, as observed in (A) second instar larvae (*n* = 5) and (B) adult beetles (*n* = 4), with results analyzed via Wilcoxon signed‐rank test, no significance.

Second‐instar larvae that were fed amphotericin B 24‐h before larval homogenization, supernatant plating, and plate incubation. After a 48‐h incubation, it was found that colony counts between the treatments were comparable, with no significant difference between treatments (Figure 2A). A similar observation was made with adult beetles (Figure 2B) and a Wilcoxon signed rank test revealed no significant difference between treatments.

**Figure 3:**
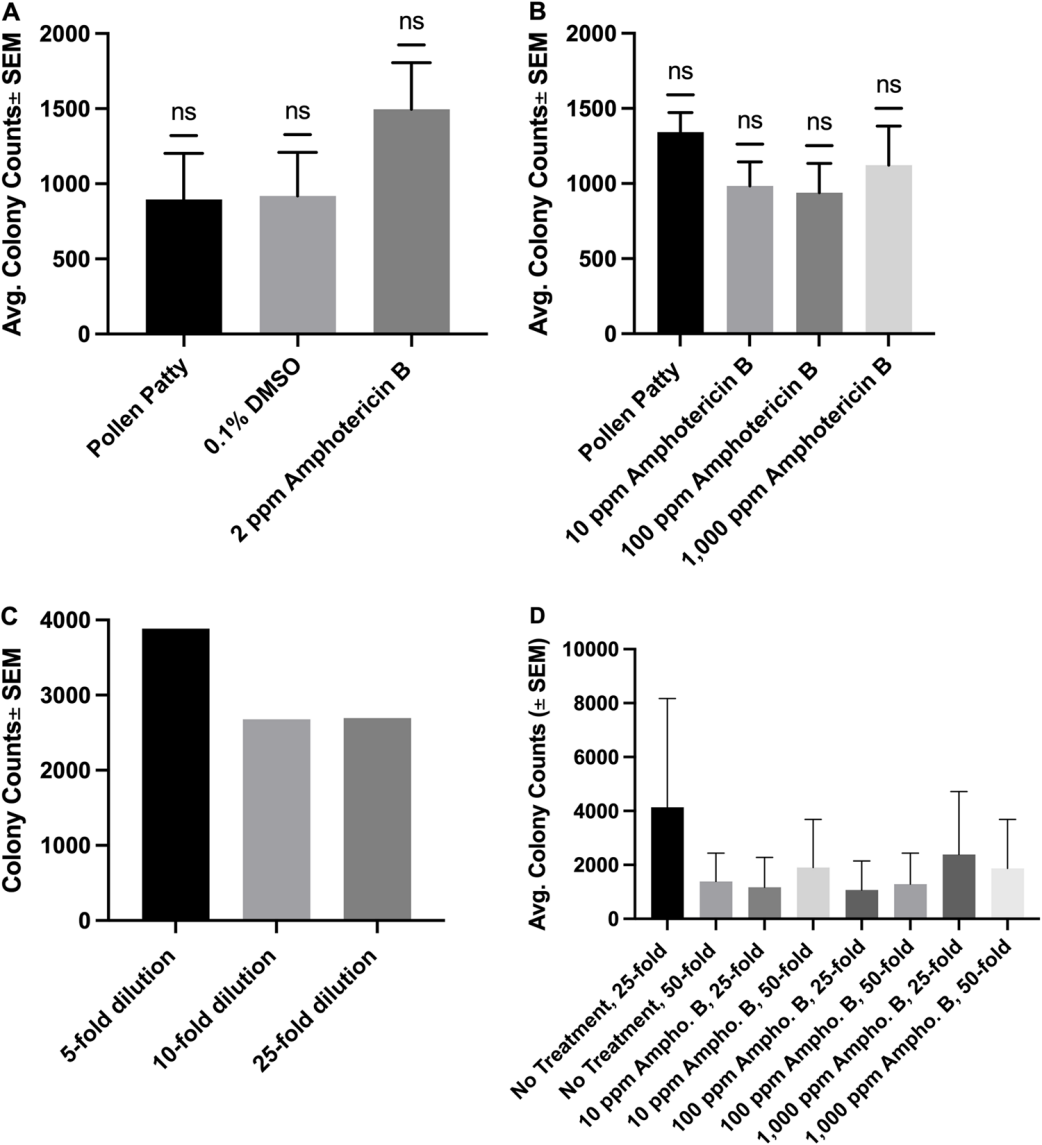
Results of fungicide feeding throughout *A. tumida* larval development through pollen patty/agarose feeding discs, starting with lower amphotericin concentrations (A; *n* = 4) and ranging to higher concentrations without use of DMSO as the vehicle control (B; *n* = 4). (C and D) Depict attempts to further dilute amphotericin B homogenate. Wilcoxon signed‐rank test revealed no significant difference (A and B), not enough reps for testing (C and D).

The results from the pollen patty/agarose disk feeding bioassays, which delivered amphotericin B for the entirety of development, were also rather perplexing. In the first set of feeding bioassay results, it was seen that 2‐ppm amphotericin B feeding treatments yielded higher colony counts than both the pollen patty agarose and 0.1% DMSO controls (Figure 3A). It was then speculated that perhaps the concentration of amphotericin B was too low to inhibit *K. ohmeri*, so the concentration was raised to a range of 10–1000 ppm, however, average colony counts were still comparable and the highest average colony counts resulted from the 1000 ppm amphotericin B treatment (Figure 3B). It was next hypothesized that perhaps the homogenate dilution (5‐fold) was too high to allow for discrimination between treatments, so dilutions of 5‐fold, 10‐fold, and 25‐fold were tested using an untreated beetle (Figure 3C). When a numeric difference was observed between the fivefold dilution had approximately 1000 more colonies than the 10‐fold and 25‐fold dilutions, the decision was made to further dilute the homogenate to 50‐fold. Subsequent tests of 25‐fold and 50‐fold dilutions of several concentrations of amphotericin B yielded somewhat stochastic results, with lower average colony counts for the 25‐fold dilutions of the 10 and 100 ppm amphotericin B treatments (Figure 3D). Comparisons of laboratory‐reared and wild caught beetles were also singularly unpredictable, as 1 or 0 colonies were observed at most 25‐fold and 50‐fold dilutions, with the notable exception of one wild caught beetle that had a colony count of 1 at the 25‐fold dilution and 883 at the 50‐fold dilution.

The injection bioassays proved to be a somewhat delicate undertaking, as determining the best way to inject beetles without killing them was somewhat difficult at first. Additionally, if the aluminum block was too cold and injection took too long the larvae could freeze. However, over nine replicates of 10 beetles per treatment, survivorship greatly increased, with mortality ranging from 0% to 30% for the first rep and from 70% to 90% by the sixth rep. Injections were most successful when carried out dorsally between the second and third thoracic segments, as abdominal injections tended to result in more cuticle tearing and hemolymph leakage. When *K. ohmeri* levels (50‐fold dilution) were tested second instar larvae directly after injection, colony counts of 6, 14, and 5 resulted from CNS buffer, 0.1% DMSO, and 1 ppm amphotericin B treatments, respectively. Conversely, injected third instar larvae allowed to feed on pollen patty for 2 days postinjection showed colony counts of 3 for the CNS buffer, 27 for 0.1% DMSO, and 26 for 1 ppm amphotericin B.

### 3.3 Pupation Bioassays

**Figure 4:**
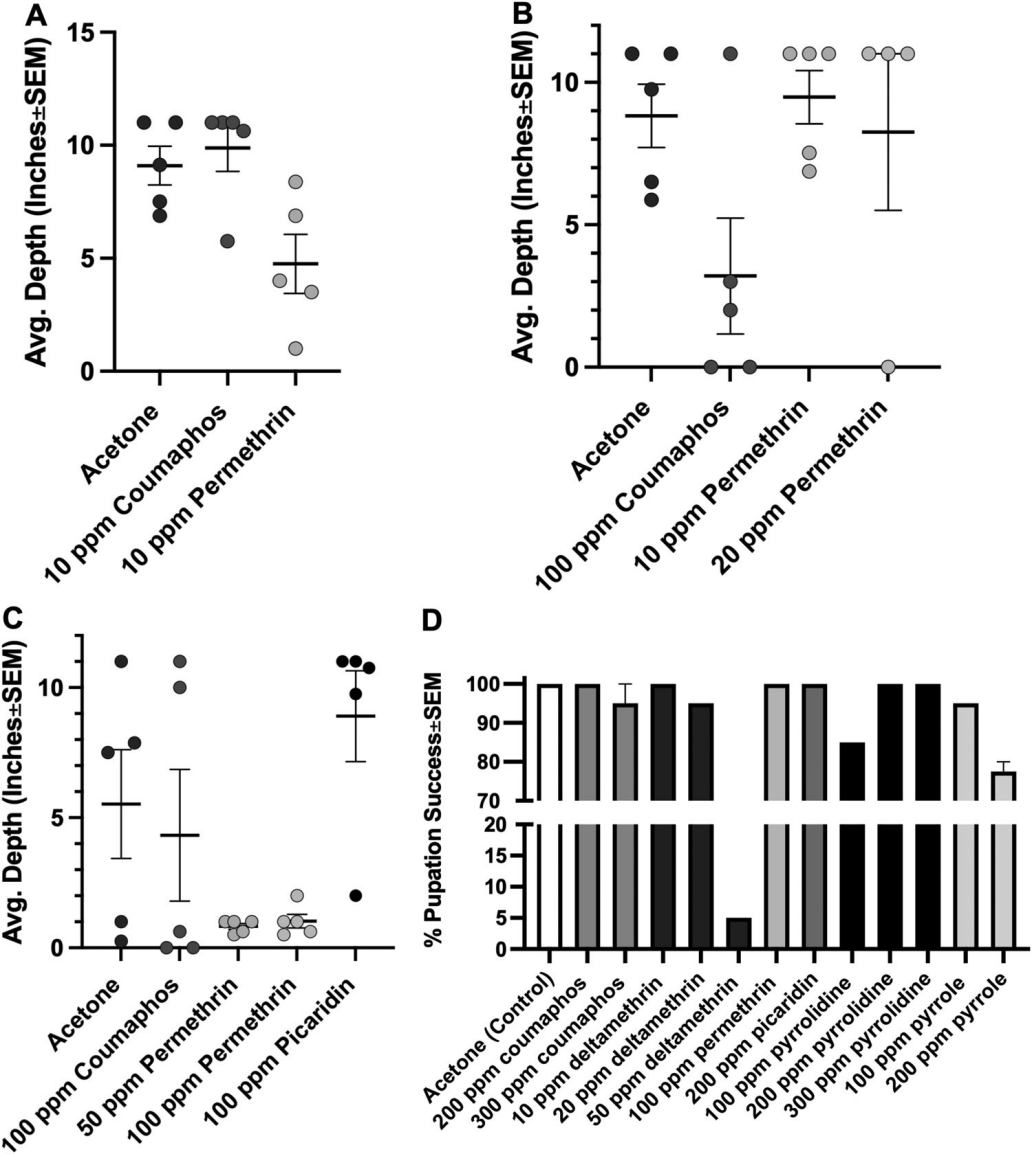
Results of pupation depth bioassays 1‐3 (A–C) and pupation success bioassays (D).

Overall, pupation depth bioassays revealed that larvae in the acetone control tended to burrow the entire 12 inches of the glass tube and into the conical below. The same deep burrowing was observed at 10 ppm coumaphos, 10–20 ppm permethrin, and 100 ppm picaridin treatments. However, at 100 ppm coumaphos burrowing depth tended to be much shallower, with an average of 3–4 in. (Figure 4A–C). Similarly, treatments of permethrin at 50 and 100 ppm essentially stopped beetles from burrowing past the treatment (Figure 4A–C). Unfortunately, several subsequent pupation depth bioassays resulted in larvae that would not burrow and essentially desiccated and died atop the soil.

Preliminary pupation success experiments have thus far appeared to be a promising pupation test method. Despite the fact that most of the control larvae from the first pupation depth experiment escaped, the results still helped inform treatments, as 85% of larvae successfully pupated beneath the 100 ppm coumaphos, whereas only 45% larvae successfully burrowed and pupated at the 100 ppm permethrin, and all larvae died in or above the 100 ppm deltamethrin treatment. The next range of treatments revealed that at 50 ppm deltamethrin 75% of larvae remained alive but were slowly desiccating and only one larva had successfully pupated. None of the other treatments, including picaridin (100–200 ppm), pyrrole (100–200 ppm), and pyrrolidine (100–300 ppm; Figure 4D) reduced pupation success to below 80% (Figure 4D). Future tests could implement the same methods to both increase the concentration of these chemicals and test new compounds.

## 4 Discussion

Understanding how insecticides impact pests is crucial to establishing optimal treatment methods, and *A. tumida* is clearly a pest to which more study needs to be devoted. Even though chemical treatments have been used over the years, these tended to be chosen based on chance and ease of availability, as seen in the use of coumaphos, a treatment that was already widely used in hives and well‐known to beekeepers (Elzen et al. 1999). However, results of this study alone indicate that coumaphos is less effective than several pyrethroids, including the common hive‐treatment *tau*‐fluvalinate. As *A. tumida* resistance to *tau*‐fluvalinate has been documented (Kanga, Marechal, Legaspi, and Haseeb 2021) it is possible that the 6‐ to 20‐fold difference in the LC_50_ values between *tau*‐fluvalinate and the other pyrethroids used in this study could be a product of resistance development in local populations from which the lab colony was collected.

Repeated exposure to similar chemistries over the years has led to organophosphate and pyrethroid resistance, which has been documented in *A. tumida* adults (Kanga, Marechal, and Ananga 2021). In one study it was demonstrated that *A. tumida* adults developed 5.4‐fold *tau*‐fluvalinate resistance and 43.7‐fold coumaphos resistance over a 10‐year time period (Kanga, Marechal, Legaspi, and Haseeb 2021). A recent study examined *A. tumida* resistance mechanisms was performed using insecticides alone and with synergists, revealing that the likely mechanism of resistance to these compounds is the activity of esterases and mixed function oxidases (Kanga, Marechal, Legaspi, and Haseeb 2021), but more work is needed to better understand *A. tumida* biology and responses to insecticide treatments. Resistance development aside, weather conditions can lessen the effectiveness of soil treatments (Levot and Haque 2006). It is also difficult to time when the majority of larvae will be exiting the hive for pupation; thus, soil treatments can be ineffective and necessitate frequent reapplication (Kanga and Somorin 2012), all of which leads to the second important difficulty in establishing effective *A. tumida* control methods: the *A. tumida* lifecycle and the uniquely adapted biology of this pest to the apiary environment.

Previous resistance studies with coumaphos and *tau*‐fluvalinate were performed using glass vial bioassays (Kanga, Marechal, Legaspi, and Haseeb 2021), but the feeding bioassays developed in this study could provide a useful way to measure oral toxicity and monitor resistance development in future studies. Although food was provided ad libitum in this study, it is possible that surviving larvae in sugar only gel pellet trials may have cannibalized their dead or dying fellow test subjects, thus obtaining some protein, and growing a bit larger. In future work, using individual larvae containers could ensure that proper sugar and protein ratios for *A. tumida* growth were achieved. *A. tumida* toxicity tests in the past have primarily performed via contact through glass vials or topical applications (Kanga, Marechal, and Ananga 2021; Kanga, Marechal, Legaspi, and Haseeb 2021; Bisrat and Jung 2020; Kanga and Somorin 2012), with one study testing a drinking bioassay (Powell et al. 2020). Further, soil bioassays have been performed in the past, especially in reference to testing lime and diatomaceous earth, which essentially work to desiccate migrating larvae (Buchholz et al. 2009; Cuthbertson et al. 2013), but, in insecticide tests, beetles have been directly exposed to treated soil, rather than having the option to burrow through soil treatments (Kanga and Somorin 2012). Pupation did appear to be disrupted in this study, as some larvae chose to remain atop the soil, eventually dying. One hypothesis for this lack of burrowing is that these larvae were potentially not ready to pupate, or moisture levels were incorrect within the system. Since these later reps occurred in the winter months, a seasonal effect could also be possible. One solution to explore in the future would be to add third instar larvae to a container with a food source and soil and wait until they moved away from the food to add them to the pupation tubes. Knowing whether nonlethal soil treatments could still disrupt pupation would be useful information to uncover, and further soil tests, especially tests of less toxic repellent compounds (Larson et al. 2021) would be a beneficial continuation of this work.

Although oral delivery and injection of amphotericin B were not consistently effective in reducing internal *K. ohmeri* growth in *A. tumida* larvae and adults in this study, continued attention should be devoted to understanding this symbiotic relationship. *Kodamaea ohmeri* is present throughout the entire *A. tumida* lifecycle, starting in egg mucilage, and it is thought that laying eggs in clutches may be to better ensure that all eggs are inoculated with *K. ohmeri* (Amos et al. 2018). Even though one laboratory study concluded that *K. ohmeri* was merely a facultative symbiont, since *A. tumida* could still successfully survive and reproduce when external *K. ohmeri* was removed via surface sterilization (Amos et al. 2019), this is far from conclusive evidence that repression *of K. ohmeri* would not eventually be harmful to *A. tumida*, especially over multiple generations or in a field setting, which is yet to be studied. Sequencing the *K. ohemri* genome revealed hexos transporter and invertase genes, which indicates that *K. ohmeri* may be aiding *A. tumida* in metabolizing sucrose, since the *A. tumida* genome was not found to contain a sucrose metabolism invertase (Tauber, Childers, and Evans 2019).

There are multiple strains of *K. ohmeri* that have been isolated from *A. tumida* (Benda et al. 2008), and this fungus has been even more widely studied in human medicine (Kanno et al. 2017). Thus, exploring the specific strains of *K. ohmeri* that may be present in *A. tumida* population, along with testing different antifungal drugs that have been used in medical settings, such as floconazole, posaconazole, 5‐flucytosine, caspofungin, micafungin, and others (Al‐Sweih et al. 2011; Tashiro et al. 2018), could help determine the optimal fungicide to deal with *K. ohmeri* in an apiary setting. While acetic, lactic, and formic acid have all been shown to inhibit *K. ohmeri* and kill *A. tumida* adults and larvae (Schäfer et al. 2009), these treatments can be difficult to apply correctly and can be detrimental to brood and hive products (Rosenkranz, Aumeier, and Ziegelmann 2010). Examining the ability of specific fungicides to target and repress *K. ohmeri* is an important future step in understanding the benefits to *A. tumida* derived by *K. ohmeri* and helping to repress the growth of this fungi in *A. mellifera* colonies.

## 5 Conclusion

*A. tumida* presence in North American apiaries has continued to increase in the past two decades (Elzen et al. 1999), and continuous development of management options is critical in facing this ongoing threat to apiary health. This study established a series of tests with which future treatment options can be tested, facilitating an ongoing study of the effects of insecticidal and fungicidal treatments on all developmental stages of *A. tumida*. Rather than relying on *V. destructor* treatments (Elzen, Westervelt, and Lucas 2004) and indiscriminately applied soil treatments and trapping (Hood 2004), treatments targeting *A. tumida* biology could be discovered and used to minimize infestations and combat the development of *A. tumida* insecticide resistance (Kanga, Marechal, Legaspi, and Haseeb 2021). By establishing standardized *A. tumida* testing methods, the daunting process of discovering effective treatments can be streamlined and management of this pest can be more efficiently accomplished.

## Author Contributions

**Morgan A. Roth:** investigation, writing–original draft, writing–review and editing, formal analysis. **Aaron D. Gross:** funding acquisition, conceptualization, investigation, supervision, project administration, writing–review and editing.

## Conflicts of Interest

The authors declare no conflicts of interest.

## Supporting information

Supporting information.

## Data Availability

The data that support the findings of this study are available from the corresponding author upon reasonable request.
