# Supplementary material for: Optimizing Feeding and Pupation Bioassays to Assess the Effects of Insecticidal and Repellent Treatments on Aethina tumida Larval Development and Pupation Success
Source: Arch Insect Biochem Physiol. 2025 Jan 21;118(1):e70023. doi: 10.1002/arch.70023 (PMC11748190; doi:10.1002/arch.70023)
Supplement: Supplementary file 1 — Supporting information. [file ARCH-118-e70023-s001.pdf]

Feeding bioassay – agarose pellets

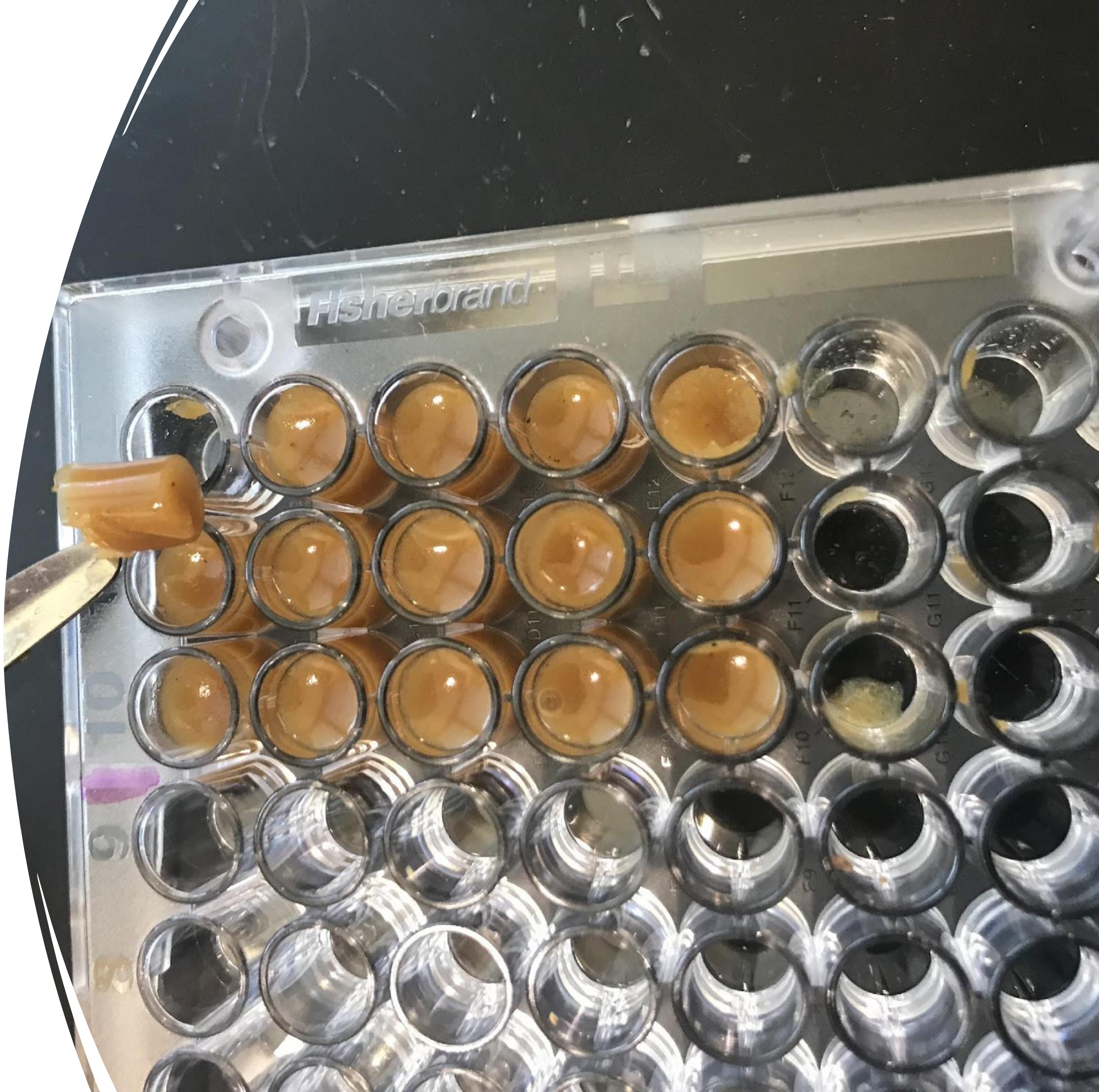

Figure S1a: Agarose feeding pellets that were optimized at 25% sugar and 2% soy protein.

Pupation depth bioassay

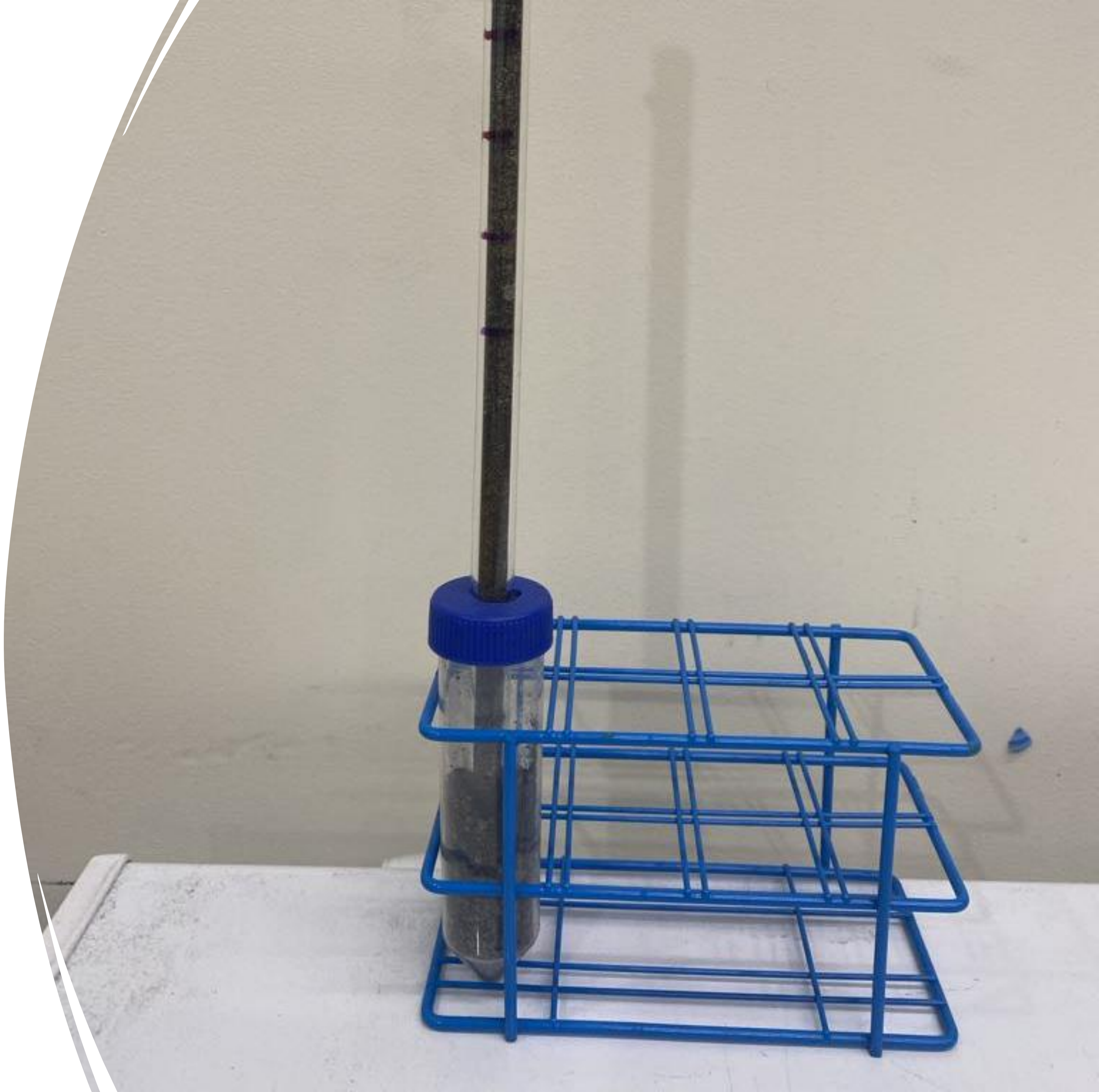

Figure S1b: Setup of the pupation depth assay.
